# Transjugular intrahepatic portosystemic shunt combined with endoscopic ultrasonography for portal vein recanalization and biopsy in malignant occlusion of the portal vein

**DOI:** 10.1055/a-2491-6852

**Published:** 2024-12-17

**Authors:** Xiaobing Wang, Feng Ding, Jun Fang, Feng Zhou, Liping Chen

**Affiliations:** 1 Department of Gastroenterology and Hepatology, Zhongnan Hospital of Wuhan University, Wuhan, China; 2 Hubei Clinical Center and Key Lab of Intestinal and Colorectal Diseases, Wuhan, China

**Fig. 1:**
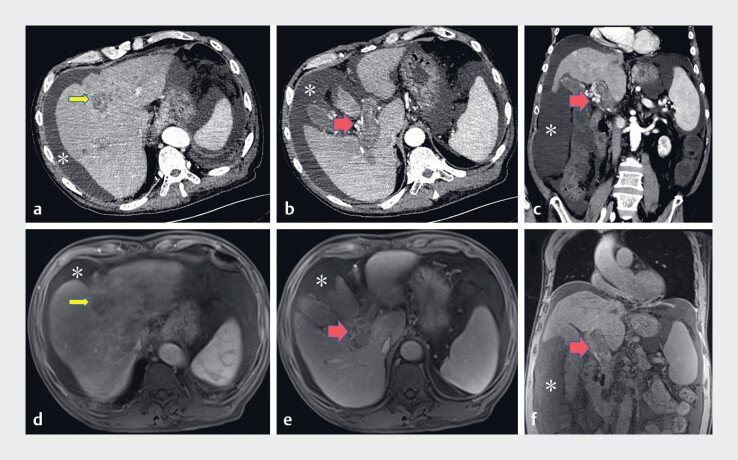
Imaging studies revealed an enhanced nodular shadow with a diameter of approximately 10 mm in the left inner lobe of the liver near the top of the diaphragm (yellow arrow), with portal vein dilation and occlusion, which was accompanied by reticular angiogenesis (red arrow), indicating cavernomatous transformation of the portal vein. Cirrhosis, splenomegaly, and obvious ascites (*) were also displayed. **a–c** Abdominal contrast-enhanced computed tomography. **d–f** Magnetic resonance imaging.

**Fig. 2:**
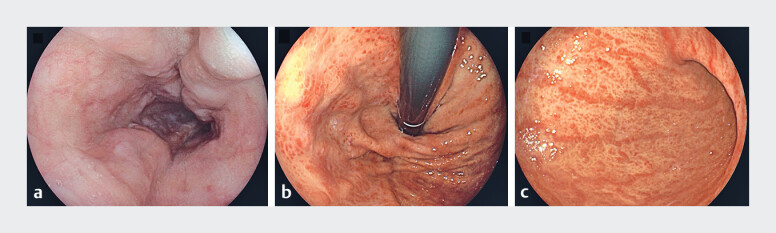
Endoscopy images. **a** Three esophageal varices. **b,c** No varices in the gastric fundus, but mucosal swelling and congestion in the gastric fundus ( **b** ) and body ( **c** ), with mosaic appearance.

A 69-year-old man presented with intermittent melena and hematemesis for 4 days, accompanied by mild abdominal pain and lower limb edema. He had a history of cerebral hemorrhage with right limb hemiplegia. Upon admission, laboratory findings revealed hemoglobin at 75.4 g/L, albumin at 29.8 g/L, blood urea nitrogen at 12.28 mmol/L, and abnormal prothrombin at 450.79 mAU/mL. Other tests were negative. Abdominal contrast-enhanced computed tomography scan and magnetic resonance imaging were conducted ( Fig. 1 ), and an upper gastrointestinal endoscopy showed esophageal varices with red sign and portal hypertensive gastropathy ( Fig. 2 ). Treatment with somatostatin and a proton pump inhibitor was initiated, but melena persisted, with a mild decrease in hemoglobin.

A multidisciplinary team discussion concluded that traditional endoscopic, surgical, interventional treatments and effective tumor treatment were unfeasible. The treatment focused on two main issues: achieving portal vein recanalization to alleviate portal hypertension and achieve hemostasis, and obtaining tumor tissues for assessment and determination of appropriate multimodal treatment. Owing to occlusion of the main portal vein and branches, along with the presence of moderate ascites, the traditional transjugular intrahepatic portosystemic shunt (TIPS) method was not viable. However, endoscopic ultrasound (EUS)-guided portal vein localization before TIPS could be a safe alternative for benign portal vein occlusion and might be adapted for this case. In addition, it seemed possible to perform EUS-guided fine-needle aspiration (FNA) biopsy during the process.

**Fig. 3:**
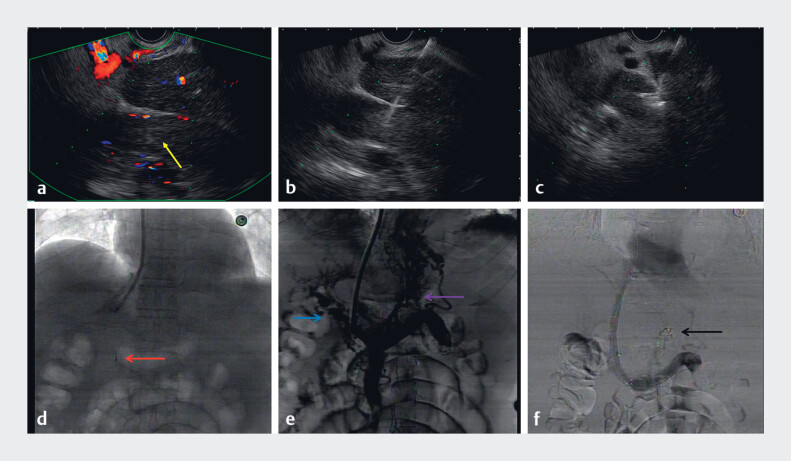
Endoscopic ultrasound (EUS) and radiography images. **a** EUS showed a hypoechoic mass in the portal vein with no flow signals (yellow arrow). **b** EUS-guided fine-needle aspiration in the portal vein using a 22-G puncture needle. **c** A coil was released in the portal vein. **d–f** Transjugular intrahepatic portosystemic shunt procedure. **d** The coil was visible under X-ray fluoroscopy. **e** Radiography presented disorganized blood vessels of cavernomatous transformation of the portal vein (blue arrow) and collateral vessels (purple arrow). **f** After embolization of collateral vessels using several coils (black arrow) and implantation of a covered self-expanding metallic stent combined with a bare stent, radiography indicated a good blood flow from the splenic vein to the heart. The portal pressure gradient decreased from 26.5 mmHg to 14.7 mmHg.

**Fig. 4:**
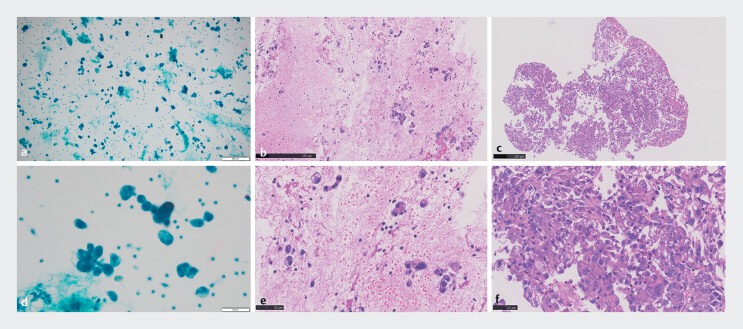
Pathology revealed poorly differentiated hepatocellular carcinoma. **a–c** Low magnification. **d–f** High magnification. **a, d** Liquid-based cytology showed cancer cells. **b, e** A small number of cancer cells were seen in the blood clot in biopsy specimens from endoscopic ultrasound-guided fine-needle aspiration. **c, f** A large number of cancer cells were visible in biopsy specimens from the transjugular intrahepatic portosystemic shunt pathway. Immunohistochemical staining indicated results of arginase-1(–), MOC31(–), GS(±)HSP70(+), glypican-3(–), hepatocyte(–)CK19(+), CK7(–), MUC1(–), Ki-67(Li:50%).

The successful attempt is shown in Video 1 and Fig. 3 . A sterilized small biopsy forceps was also used to enter the occluded portal vein to obtain specimens though a RUPS-100 channel. The pathological diagnosis was poorly differentiated hepatocellular carcinoma ( Fig. 4 ). Melena ceased by the second day, and the patient was discharged a week later.

The transjugular intrahepatic portosystemic shunt procedure combined with endoscopic ultrasound for portal vein recanalization and biopsy in malignant occlusion of the portal vein.Video 1

Portal vein occlusion with cavernomatous transformation of the portal vein presented clinical challenges, and distinguishing the tumor from thrombus was also difficult. For malignant portal vein occlusion causing portal hypertension symptoms, TIPS after EUS-guided portal vein localization, combined with EUS-FNA biopsy, might be useful for comprehensive treatment.

Endoscopy_UCTN_Code_TTT_1AS_2AG

